# Earth-Abundant Oxygen Evolution Catalysts Coupled onto ZnO Nanowire Arrays for Efficient Photoelectrochemical Water Cleavage

**DOI:** 10.1002/chem.201403067

**Published:** 2014-08-22

**Authors:** Chaoran Jiang, Savio J A Moniz, Majeda Khraisheh, Junwang Tang

**Affiliations:** [a] Department of Chemical Engineering, University College London Torrington Place, London, WC1E 7JE (UK) E-mail: junwang.tang@ucl.ac.uk; [b] Department of Chemical Engineering, Qatar University University Road, PO Box 2713 Doha (Qatar)

**Keywords:** electrochemistry, nanostructures, photochemistry, thin films, water splitting

## Abstract

ZnO has long been considered as a model UV-driven photoanode for photoelectrochemical water splitting, but its performance has been limited by fast charge-carrier recombination, extremely poor stability in aqueous solution, and slow kinetics of water oxidation. These issues were addressed by applying a strategy of optimization and passivation of hydrothermally grown 1D ZnO nanowire arrays. The length and diameter of bare ZnO nanowires were optimized by varying the growth time and precursor concentration to achieve optimal photoelectrochemical performance. The addition of earth-abundant cobalt phosphate (Co-Pi) and nickel borate (Ni-B) oxygen evolution catalysts onto ZnO nanowires resulted in substantial cathodic shifts in onset potential to as low as about 0.3 V versus the reversible hydrogen electrode (RHE) for Ni-B/ZnO, for which a maximum photocurrent density of 1.1 mA cm^−2^ at 0.9 V (vs. RHE) with applied bias photon-to-current efficiency of 0.4 % and an unprecedented near-unity incident photon-to-current efficiency at 370 nm. In addition the potential required for saturated photocurrent was dramatically reduced from 1.6 to 0.9 V versus RHE. Furthermore, the stability of these ZnO nanowires was significantly enhanced by using Ni-B compared to Co-Pi due to its superior chemical robustness, and it thus has additional functionality as a stable protecting layer on the ZnO surface. These remarkable enhancements in both photocatalytic activity and stability directly address the current severe limitations in the use of ZnO-based photoelectrodes for water-splitting applications, and can be applied to other photoanodes for efficient solar-driven fuel synthesis.

## Introduction

The development of efficient methods for generating clean and sustainable energy is critically important to limit harmful greenhouse-gas emissions from burning of fossil fuels and to meet the rapid increase in global energy demand. Therefore, considerable research has been conducted, spanning several decades, to find alternative, clean, and more efficient energy resources and conversion pathways to replace finite resources such as fossil fuels.[1] Artificial photosynthesis (water splitting) is an attractive approach to meet current targets to efficiently and inexpensively convert solar energy to a storable and transportable form of energy. Splitting of water by direct sunlight into molecular oxygen and hydrogen in a photoelectrochemical (PEC) cell is one such promising method to produce a chemical fuel (hydrogen) that can be utilized in a fuel cell or by direct combustion.[2] Of the two half-reactions, water oxidation is widely considered to be more challenging, given the fact that generation of one molecule of O_2_ requires four holes, generated on a timescale five orders of magnitude slower than the reduction reaction.[3] Therefore, the search for stable, efficient water-oxidation photocatalysts is widely regarded to be significant for large-scale water photolysis. Since the discovery of TiO_2_ as a stable photoanode for photoelectrochemical water cleavage by Honda and Fujishima in 1972,[4] considerable efforts have been made to seek an efficient and stable photoanode for water oxidation. Studies have focused on semiconductor metal oxides such as TiO_2_,[5] ZnO,[6] WO_3_,[7] BiVO_4_,[8], Fe_2_O_3_,[9] and Ag_3_PO_4_.[3a] However overcoming their poor stabilities and/or poor utilization of solar energy still remains a significant challenge. Furthermore, the efficiency of these semiconductor photocatalysts is seriously limited by factors including, but not limited to, low charge-carrier mobility, poor conductivity, low rates of surface reactions, and high charge-carrier recombination. Studies have shown that photogenerated electrons and holes recombine rapidly, on the scale of nano-microseconds for colloidal TiO_2_,[3b,^10^] and on the order of picoseconds for α-Fe_2_O_3_.[11] Despite this, carrier recombination could be alleviated by applying a small external bias to the light-absorbing photoanode to provide sufficient overpotential to transfer electrons to the counter electrode in a PEC. Zinc oxide (ZnO), with a bandgap energy of 3.2 eV, has been reported to be a suitable model semiconductor for solar water oxidation due to its low onset potential[6] and high electron mobility. The latter is several orders of magnitude higher than that of TiO_2_, and thus its electrical resistance is lower and its electron-transfer efficiency higher.[12] However, the drawbacks of utilizing ZnO include low hole mobility[13] and slow kinetics at the ZnO/electrolyte interface, which result in fast electron–hole recombination and thus limit the overall applied bias photon-to-current efficiency (ABPE). Additionally, the extremely poor photostability of ZnO in aqueous solution limits the performance of ZnO-based photoanodes significantly, and has restricted their widespread employment in commercial devices.[14] To date, numerous strategies have been developed to overcome its poor activity, including 1) fabricating a multisemiconductor system (e.g., Si/ZnO core/shell nanowires) to reduce hole–electron recombination,[15] 2) constructing 1D nanostructured ZnO-based electrodes with various morphologies (e.g., nanotubes,[16] nanorods/nanowires[17]) for increased surface area and improved charge transport and light trapping, and 3) loading of oxygen-evolution catalysts (OECs) such as cobalt phosphate (Co-Pi) to improve the electron–hole separation and O_2_ evolution kinetics.[18] To address the issue of poor stability, it has been demonstrated that a thin layer of SnO_2_ can act as a partial passivating layer for ZnO nanowires.[19] However, in all cases the ABPE (*η*) and incident photon-to-current conversion efficiency (IPCE) are still moderate, for example, nitrogen-doped ZnO (*η*=0.35 %, IPCE=35 % at 350 nm)[20] and Si/ZnO core/shell nanowires (*η*=0.38 %).[15] The poor stability of the photoanode still remains a significant challenge.

In our material-design strategy, employing a 1D nanostructured morphology for ZnO offers the potential advantage of improved charge transport over a flat surface, while simultaneously suppressing light scattering due to the light-trapping effect.[12a, 21] Furthermore, the optimization of their length and diameter will maximize light absorption and provide a short charge-carrier diffusion length, and the use of cheap, earth-abundant OECs on the surface of ZnO nanowires could improve the photoanodic performance by their acting as hole-trapping sites for water oxidation and in situ charge separation. We also expect that employing high surface area ZnO nanowires would result in significantly increased cocatalyst deposition on the ZnO surface compared to flat ZnO films. Therefore, to directly address the aforementioned challenges in the employment of ZnO photoanodes, we herein report the morphological optimization of well-aligned 1D ZnO nanowire arrays. The loading of water-oxidation catalysts [Co-Pi and nickel borate (Ni-B)] on ZnO for efficient photoelectrochemical water splitting was investigated. Finally, the highest IPCE recorded for ZnO-based photoelectrodes and unprecedented stability were achieved.

## Results and Discussion

**Figure 1:**
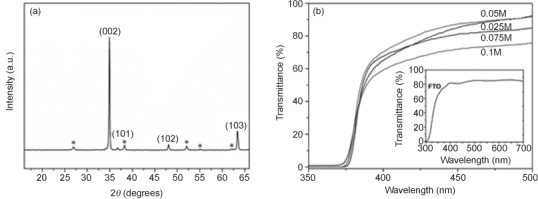
a) XRD pattern of ZnO nanowire array grown on FTO glass substrate at 90 °C for 4 h. Asterisks indicate peaks of SnO_2_ (FTO substrate). b) UV/Vis transmittance spectra of ZnO films fabricated at 90 °C for 4 h as a function of precursor concentration. The inset shows the transmittance spectrum of a bare FTO glass substrate.

The effect of deposition time and the concentration of precursor were investigated for growth of bare ZnO nanowire arrays. XRD (Figure 1 a) revealed that the as-prepared ZnO film has a wurtzite structure, with the strongest ZnO (002) peak indicating strong preferred orientation in the *c*-axis direction, as expected.[22]

The UV/Vis transmittance spectra and SEM images of bare ZnO films prepared with different reaction times are shown in Figures S1 and S2 (Supporting Information). All films exhibited good transparency in the visible range (380–500 nm); for example, the sample grown for 1 h displayed close to 90 % transmittance in this wavelength range. The bandgap energy of ZnO nanowires was estimated to be about 3.3 eV from the UV/Vis absorption spectra (Supporting Information Figure S1, inset), and as expected, did not vary with reaction time. The SEM images obtained for bare ZnO nanowires synthesized with varying deposition time (Supporting Information Figure S2) clearly show that all nanowires had an average diameter of about 50 nm independent of deposition time, whereas their lengths increased with time, similar to previous reports.[22b] However, only a slight increase in length was observed when the reaction time increased to 5 h, which indicated that the dissolution/precipitation equilibrium was attained.[23] Figure S3 (Supporting Information) shows the *I*–*V* curves for ZnO films prepared with 0.025 m precursor at 90 °C as a function of growth time in 0.2 m Na_2_SO_4_ with phosphate buffer (pH 7) electrolyte under 100 mW cm^−2^ illumination. The dark current was negligible over the entire potential range from 0.2 to 1.6 V (vs. RHE). The maximum photocurrent is strongly dependent on the length of the nanowires. Longer nanowires maximize the light absorption and provide more reaction sites, and thus higher photocurrent. ZnO nanowires prepared at 4 and 5 h, which had similar lengths of about 1300 nm, both resulted in similarly high photocurrents.

**Figure 2:**
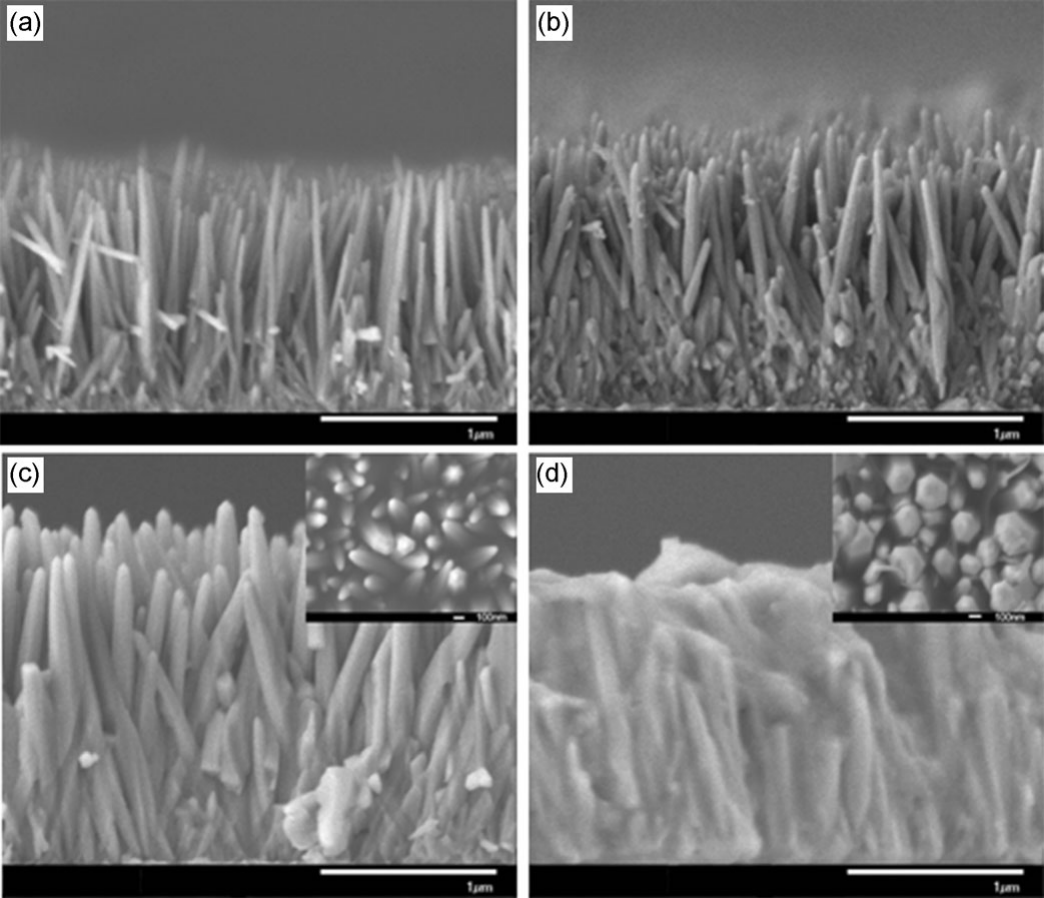
SEM images of ZnO nanowire arrays grown by hydrolysis/condensation reaction at 90 °C for 4 h as a function of precursor concentration. a) 0.025, b) 0.05, c) 0.075 m, and d) 0.1 m. Insets: top-view SEM images.

**Figure 3:**
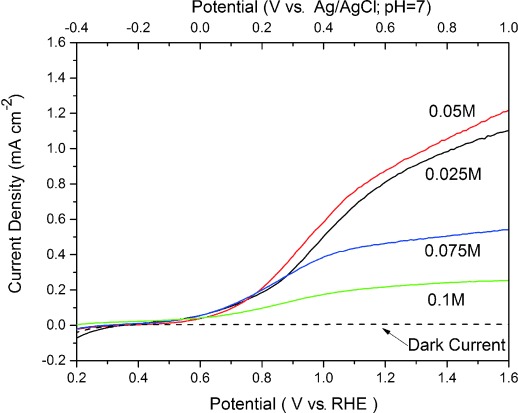
*I*–*V* curves measured in a 0.2 m Na_2_SO_4_ solution with phosphate buffer (pH 7) for ZnO films synthesized at 90 °C for 4 h with varying precursor concentration.

Next, we investigated the effect of precursor concentration while keeping the reaction time fixed (4 h). Table 1 summarizes the effect of precursor concentration on the length and diameter of the nanowires. Both the average length and diameter increased as a function of precursor concentration, the length from 1300 nm (0.025 m) to 1800 nm (0.075 m) and the diameter from about 50 to about 110 nm (Figure 2). However, SEM images of ZnO nanowires grown with a zinc nitrate concentration of 0.1 m show a significant morphology change to a mixture of nanowires and nanoflakes that results in a condensed structure (1500 nm length). The UV/Vis transmittance spectra of ZnO films as a function of precursor concentration are shown in Figure 1 b. As a reference, the transmittance spectrum of an uncoated fluorine-doped tin oxide (FTO) glass substrate was recorded (Figure 1 b inset). As expected, all ZnO films showed closed to zero transmittance in the UV range and exhibited no variation in bandgap (≈3.3 eV). The *I*–*V* curves of ZnO nanowires prepared at 90 °C for 4 h with various precursor concentrations (Figure 3) indicate the dramatic effect of nanowire length and diameter on the photocurrent. Since ZnO-based semiconductors have a high electron mobility (ca. 400 cm^2^ V^−1^ s^−1^ at 300 K)[24] but low hole mobility (1–15 cm^2^ V^−1^ s^−1^ at 300 K),[25] efficient charge-carrier separation and activity are highly dependent on the hole diffusion length. Although longer and wider nanowires can absorb more photons, as indicated by the UV/Vis absorption spectra, and likely provide a larger surface area, a larger diameter also leads to a longer hole diffusion length, which would increase charge recombination and then lower reactivity. Therefore, the best-performing ZnO nanowire arrays should have optimized length and diameter to balance these key factors related to the photoreaction.

**Table 1:** Effect of precursor concentration *c* on the length *l* and diameter *d* of the ZnO nanowires

*c* [mol L^−1^]	*l* [nm]	*d* [nm]
0.025	1300	50
0.05	1400	70
0.075	1800	110
0.1	1500	250

Our optimization procedure resulted in a ZnO nanowire array with a length of 1400 nm and diameter of 70 nm prepared with 0.05 m precursor concentration at 90 °C for 4 h, which gave a photocurrent density of 0.62 mA cm^−2^ at 1 V (vs. RHE) and highest photocurrent density of 1.2 mA cm^−2^ at 1.6 V (vs. RHE) due to its optimized surface area, light absorption, and hole diffusion length, which is much higher than the photocurrent density of bare ZnO nanowires reported recently (0.4 mA cm^−2^ at 1.0 V vs. RHE).[26]

**Figure 4:**
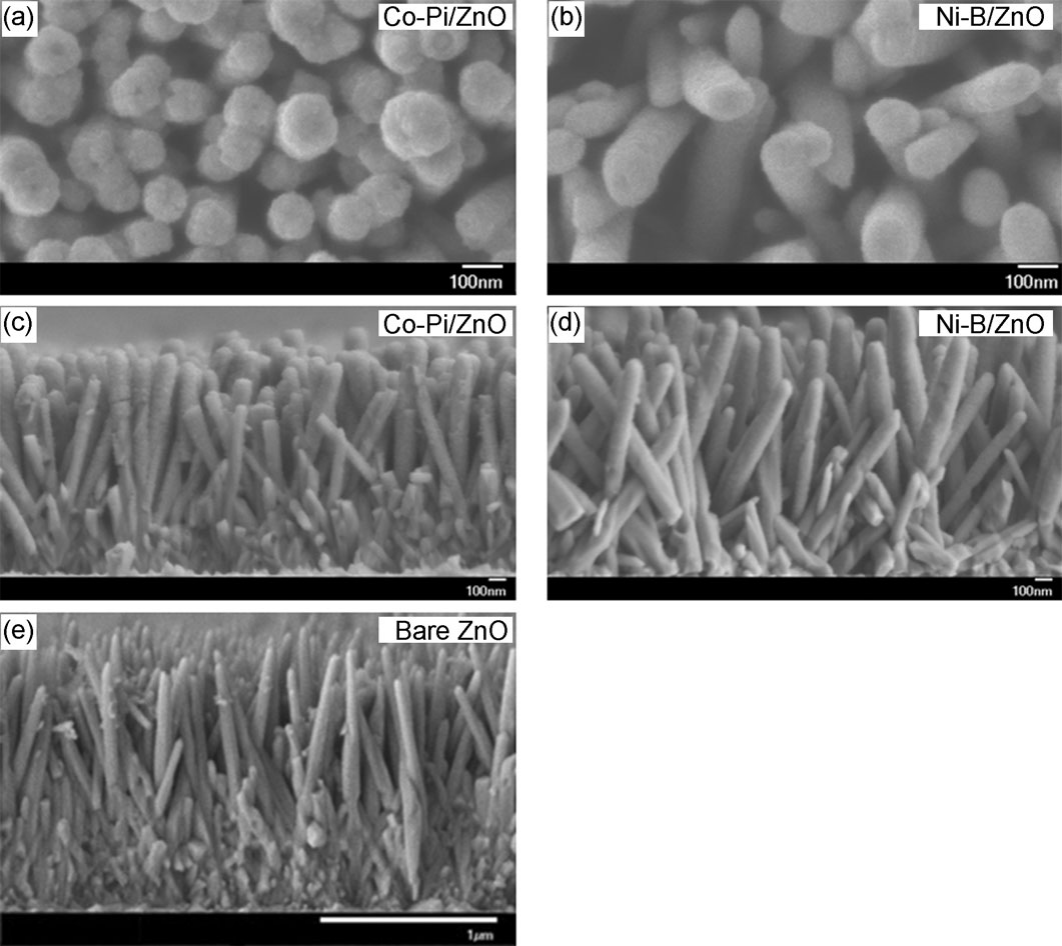
Typical SEM images of ZnO nanowires before PEC measurement. a) Top view of Co-Pi/ZnO. b) Top view of Ni-B/ZnO. c) Side view of Co-Pi/ZnO. d) Side view of Ni-B/ZnO. e) Side view of bare ZnO.

To improve the kinetics for water oxidation and electron–hole separation, the OECs Co-Pi and Ni-B were deposited on the optimized ZnO nanowire arrays by a simple photoassisted electrodeposition procedure.[27, 28] Figure 4 shows typical SEM images of Co-Pi/ZnO and Ni-B/ZnO films before PEC tests, as well as that of a bare ZnO film (Figure 4 e, prepared with 0.05 m precursor concentration at 90 °C for 4 h) for comparison. Deposition of Co-Pi or Ni-B on the ZnO surface resulted in uniform coverage along the entire length of the nanowires. In addition, compared with bare ZnO nanowires, the average length of the Co-Pi/ZnO and Ni-B/ZnO nanowires is maintained, but their average diameter increased slightly from 70 nm for bare ZnO to 120 nm and 100 nm for Co-Pi/ZnO and Ni-B/ZnO nanowires, respectively, which is attributed to addition of the catalyst layer and formation of catalyst/ZnO core/shell-type arrays.

**Figure 5:**
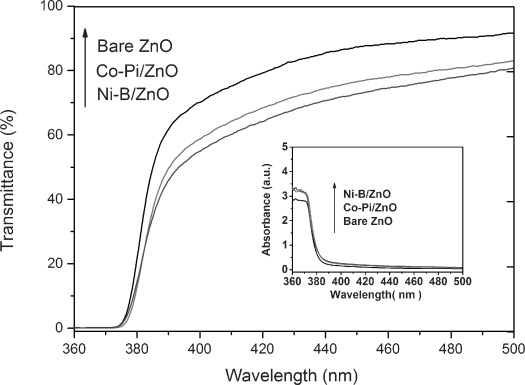
UV/Vis transmittance spectra of bare ZnO, Co-Pi/ZnO, and Ni-B/ZnO. Inset: corresponding absorption spectra.

The UV/Vis transmittance spectra of optimized bare ZnO and cocatalyst-modified ZnO films are shown in Figure 5. All ZnO-based films exhibited good transparency in the visible range but almost zero transmittance in the UV range. The bandgap energy *E*_g_ of as-prepared ZnO films was estimated from the absorption spectra. (Figure 5 inset).[23] The *E*_g_ value was calculated to be about 3.3 eV for both bare and cocatalyst-modified ZnO films; thus, addition of cocatalysts did not alter the bandgap or light absorption of ZnO significantly.

**Figure 6:**
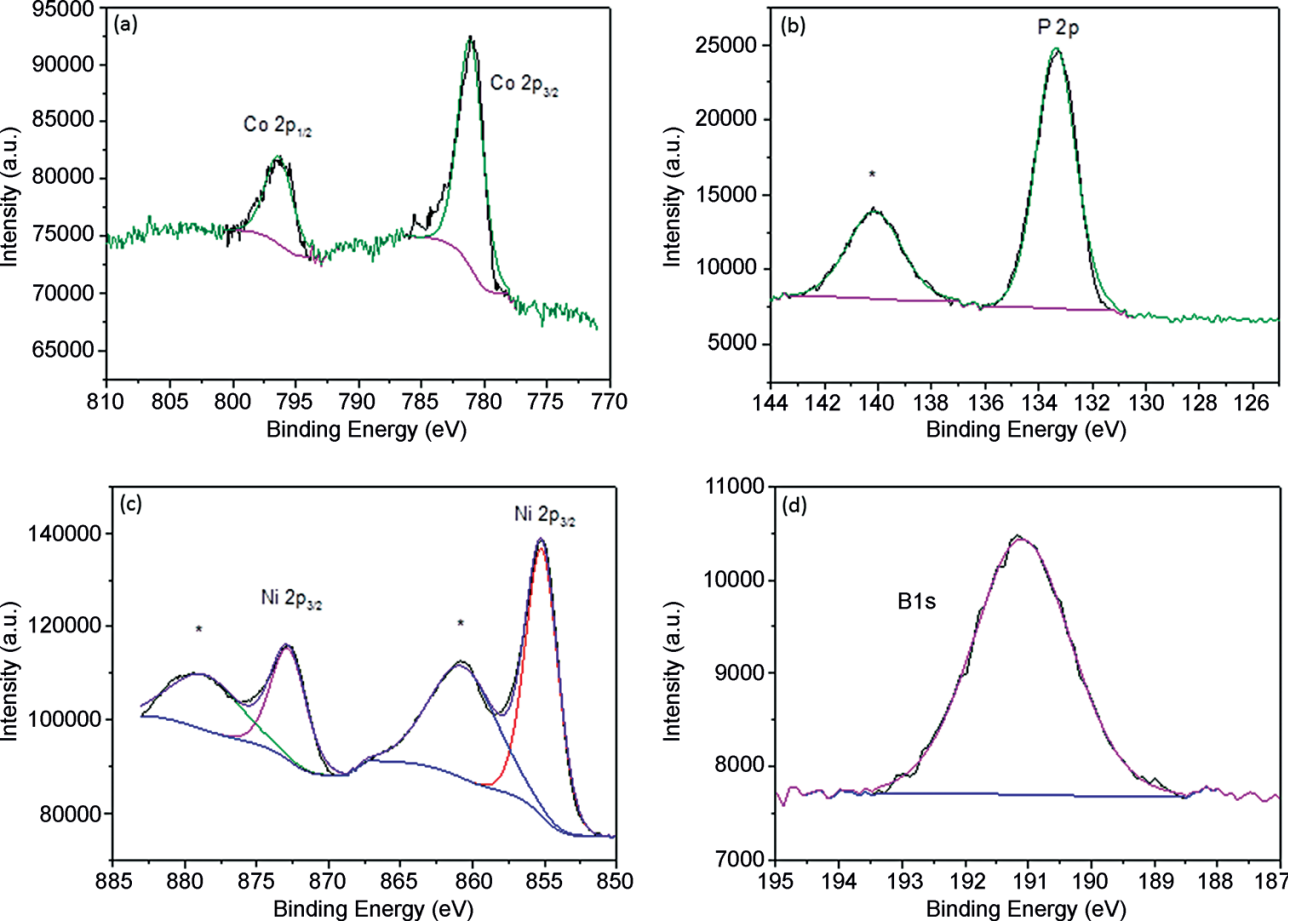
XP spectra of a) Co 2p, b) P 2p, c) Ni 2p, and d) B 1s.

High-resolution X-ray photoelectron spectroscopy (XPS) was used to ascertain the presence and exact valence states of the elements of the cocatalyst-loaded ZnO nanowire arrays. In all cases Zn 2p peaks were seen at 1022 and 1045 eV due to Zn^2+^ (Supporting information Figure S4). For Co-Pi-loaded ZnO, the presence of the Co 2p peaks at 781.1 and 796.3 eV are in good agreement with those previously reported for Co-Pi OECs loaded on the surface of semiconductors (Figure 6 a).[29] Furthermore, the P 2p peak was observed at 133.4 eV, but in addition another P peak was observed at 140.1 eV (marked with a star), which is characteristic of P absorption on defect states in ZnO but is not part of the OEC (Figure 6 b).[30] However, due to peak overlap, it is possible that this could be due to contributions from the Zn 3s peak and the Sn 4s peak (from the FTO substrate), both commonly found in the same region, as we also observed a similar peak in the XPS spectrum of bare ZnO (Supporting Information Figure S4b). For Ni-B-loaded ZnO, two clear Ni 2p peaks were observed at 855.2 and 872.8 eV, which likely correspond to Ni^2+^ or Ni^3+^ (Figure 6 c); however, as the binding energies of these two states suffer from a high degree of overlap, it is difficult to distinguish the exact nature of Ni with confidence.[31] The two corresponding Ni 2p satellite peaks (marked with stars) were also found, at 860.7 and 878.6 eV, respectively. For boron, the expected singlet peak was found at 191.1 eV (Figure 6 d), indicative of a B^3+^ environment and in agreement with recently reported XPS spectra of Ni-B catalysts.[28]

**Figure 7:**
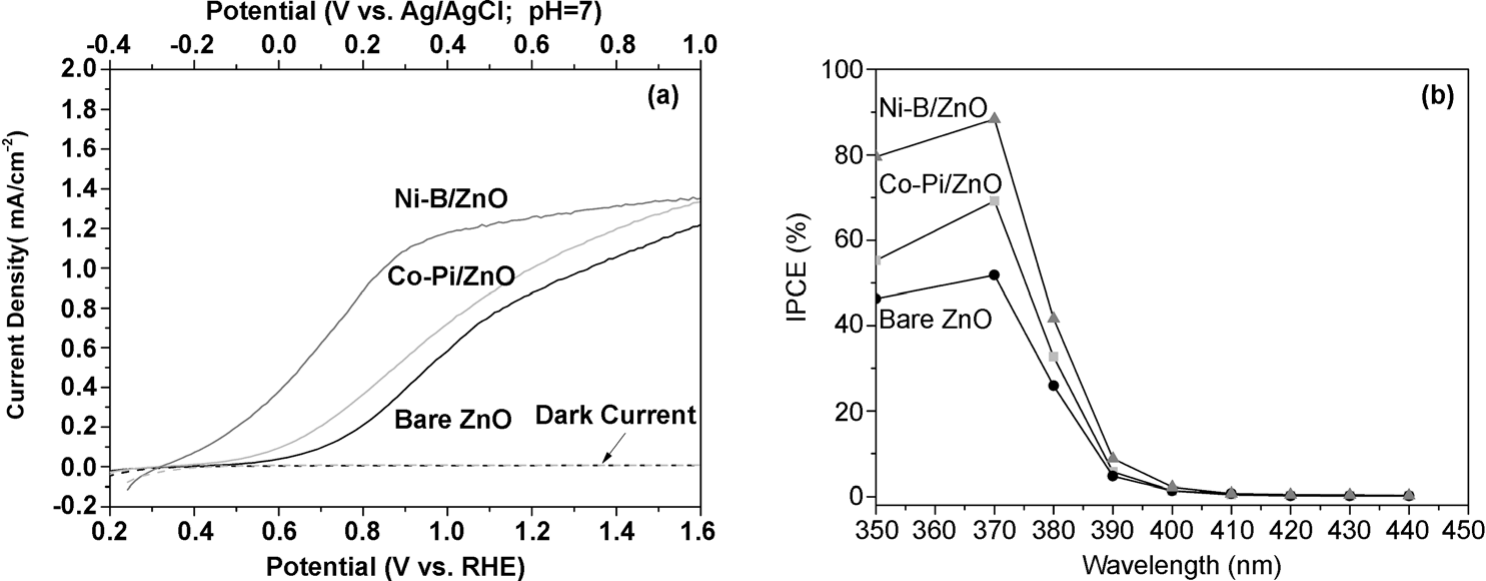
a) *I*–*V* curves of bare ZnO, Co-Pi ZnO, and Ni-B/ZnO films. b) IPCE spectra of bare ZnO, Co-Pi/ZnO, and Ni-B/ZnO films.

Figure 7 a shows the *I*–*V* curves of optimized ZnO (length 1400 nm, diameter 70 nm), Co-Pi/ZnO, and Ni-B/ZnO photoelectrodes in sodium sulfate or potassium borate electrolyte. In comparison with bare ZnO, which has an onset potential of 0.5 V (vs. RHE), the photocurrent onset is cathodically shifted by about 0.1 V (vs. RHE) for Co-Pi-modified ZnO, which is attributed to the Co-Pi catalyst mitigating hole–electron recombination by acting as a hole-trapping site to increase charge-separation efficiency. Surprisingly, the onset potential for Ni-B/ZnO is shifted cathodically by as much as 0.2 V compared to bare ZnO to about 0.3 V (vs. RHE). A low onset potential is crucial for widening the operating window and therefore achieving a high ABPE. Similar to the Co-Pi cocatalyst, Ni-B also appears to act as a hole-trapping site that can facilitate hole transfer on the semiconductor surface and thus decrease electron–hole recombination. The overall photocurrent density is also increased by loading of Co-Pi and Ni-B onto ZnO, which results in photocurrent densities of 0.72 and 1.22 mA cm^−2^ at 1.0 V (vs. RHE) for Co-Pi/ZnO and Ni-B/ZnO, respectively. More interestingly, a saturated photocurrent density of about 1.1 mA/ cm^2^ was achieved at 0.9 V (vs. RHE) for Ni-B/ZnO instead of 1.6 V (vs. RHE) for both bare ZnO and Co-Pi/ZnO nanowires.

Recent studies have reported that the deposition of Co-Pi OEC onto n-type semiconductors such as Fe_2_O_3_,[27, 32] BiVO_4_,[33] WO_3_,[34] n-type silicon (n-Si),[35] and ZnO[30] can enhance the PEC performance under neutral conditions, because the Co-Pi complex functions as a hole-trapping site for in situ charge separation, and thus reduces surface recombination and improves the kinetics for water oxidation. For example, Zhong et al. reported a simple photoassisted electrodeposition strategy to deposit Co-Pi OEC on α-Fe_2_O_3_ (hematite) photoanodes in an aqueous, neutral phosphate medium containing Co^2+^ ions.[27] In this process, photogenerated holes from hematite are used to oxidize Co^2+^ to Co^3+^, which results in deposition of Co-Pi on the surface of the semiconductor. Ni-B has a similar catalytic mechanism for water oxidation to Co-Pi. The electron–hole pairs generated by the light-absorbing semiconductor are separated by an internal electric field,[27] and conduction-band (CB) electrons move towards to the back contact (FTO substrate), through the external circuit to the Pt counter electrode under external bias (for hydrogen evolution). Photogenerated holes migrate to the Ni-B surface and oxidize Ni from 2+ to 3+, and then O_2_ evolution occurs by withdrawal of electrons from water, accompanied by a change in Ni oxidation state back to 2+. On the other hand, it has recently been reported that the active site in the Ni-B catalyst has an intermediate oxidation state of 3.6, which indicates that the Ni center is in a formal oxidation state of 4+.[36]

To compare the light-conversion efficiency of bare ZnO and cocatalyst-modified ZnO, IPCE spectra were measured for all samples (Figure 7 b). The IPCE of optimized bare ZnO nanowires (1400 nm length) is, as expected, near-zero in the visible region (400–440 nm) but increases rapidly to about 52 % at 370 nm, which is consistent with its UV/Vis spectrum and high photocurrent, as well as being much higher than that reported for ZnO nanowire arrays in the literature (≈40 %).[6, 26, 37] Both Co-Pi and Ni-B deposits on ZnO result in a sharp increase in IPCE; Co-Pi/ZnO has a dramatically increased IPCE of about 72 % at 370 nm, and Ni-B/ZnO an even higher IPCE of about 90 % at 370 nm, which represents an increase of nearly 75 % compared to the bare ZnO film. This observation provides further explanation for the somewhat early attainment of the saturated photocurrent density (1.1 mA cm^-2^ at ca. 0.9 V vs. RHE) observed in Figure 7 a. In general, an efficient water-splitting process is highly dependent on three factors: efficient light absorption by the photocatalyst, efficient charge-carrier separation, and efficient surface reaction (charge utilization). The saturated photocurrent is observed for Ni-B/ZnO at an early stage due to efficient hole trapping and subsequent faster surface reaction compared to both Co-Pi/ZnO and bare ZnO. ZnO has an intrinsically high electron mobility[24] but very low hole mobility,[25] which leads to fast recombination; therefore, bare ZnO requires a higher electrical bias to obtain a saturated photocurrent. The early saturated photocurrent observed for Ni-B/ZnO but not Co-Pi/ZnO strongly indicates that Ni-B is a much more efficient surface cocatalyst and hole acceptor than Co-Pi. The high IPCE of Ni-B/ZnO can be attributed to increased light trapping by ZnO nanowires, efficient separation of photogenerated electrons and holes through loading with an improved surface OEC, fast rectifying electron transport through 1D ZnO nanowires to the counter electrode, and efficient surface catalysis. Furthermore the ABPE of Ni-B/ZnO was measured to be 0.4 %, which is considerably higher than recently reported values for nitrogen-doped ZnO nanowires (0.15 %),[6] Si/ZnO core/shell nanowires (0.38 %),[15] and even some visible-light-driven semiconductor-based photoanodes, such as WO_3_/C_3_N_4_/CoO_*x*_,[38] However, it is lower than that of the recently reported benchmark Co-Pi/W:BiVO_4_ photoelectrode.[39a] Thus, a new visible-light-driven junction based on 1-D ZnO, such as C_3_N_4_ is underway for more efficient utilization of solar energy.[39b]

**Figure 8:**
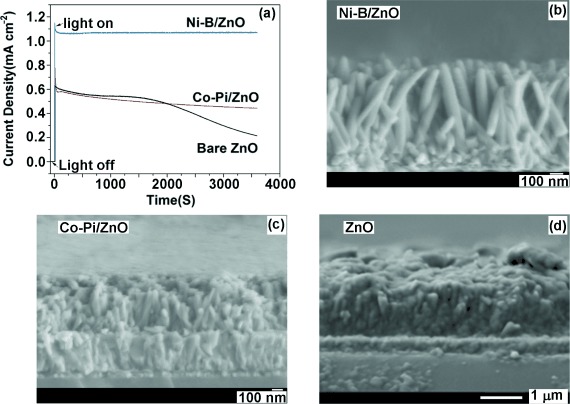
a) Current density–time curves of bare ZnO, Co-Pi/ZnO, and Ni-B/ZnO photoelectrodes measured at 1.0 V (vs. RHE) for 1 h. b)–d) Typical side-on SEM images of Ni-B/ZnO, Co-Pi/ZnO and bare ZnO nanowire arrays, respectively, after 1 h of PEC measurements.

An important consideration in the employment of photoanodes for water cleavage on a commercial scale is their stability under prolonged illumination in aqueous solution, which is a problem for ZnO-based photoanodes. Therefore, the stabilities of ZnO, Co-Pi/ZnO, and Ni-B/ZnO were investigated at a potential of 1.0 V (vs. RHE) for 1 h (Figure 8 a). For bare ZnO nanowires, very poor stability was observed with continuous decay of photocurrent; only 34 % residual photocurrent remained at the end of the experiment. A slight improvement in stability was observed for Co-Pi/ZnO, as Co-Pi itself appears to have some short-term stability in solution, followed by a relatively stable stage (65 % photocurrent remaining at the end of the experiment). This is most likely due to facile exchange of cobalt and phosphorus ions directly between the film and solution.[40] Most significantly, Ni-B/ZnO exhibited unprecedented retention of photocurrent over 1 h, which, to the best of our knowledge, is the first such observation for ZnO photoelectrodes. Similarly, a recent report also mentioned that a nickel film deposited by electron beam evaporation on an otherwise low-stability n-Si photoanode results in an unprecedented improvement in stability during PEC measurements in aqueous solution and a major shift in onset potential on immersion in potassium borate electrolyte.[31]

To confirm the improved stability of our Ni-B/ZnO junction, the morphologies of these ZnO-based photoelectrodes were examined by SEM after prolonged PEC testing (Figure 8 b–d). In agreement with the stability test, no photocorrosion was observed for Ni-B/ZnO, but the images of the bare ZnO and Co-Pi/ZnO electrodes revealed significant transformation of the structures into dense, compact films with very few distinct features and, significantly, they no longer resembled nanowire arrays. The side-on views of these samples before and after PEC testing clearly show the effect of photocorrosion on their morphologies. Only Ni-B/ZnO retains the vertically aligned nanowire morphology (Figure 4 and Figure 8 b–d). Furthermore, XRD analysis of the ZnO and Co-Pi/ZnO photoelectrodes after water-oxidation experiments (Figure S5, Supporting Information) revealed the emergence of a Zn_3_(PO_4_)_2_ phase (JCPDS card No. 33-1474) due to reaction with phosphate ions and is consistent with ZnO photocorrosion.[41] Similarly, high-resolution XPS analysis of these samples (Supporting Information Figure S6) revealed that the O 1s peak of bare ZnO shifted to 531.3 eV with only a small contribution from ZnO at 529.7 eV, whereas extreme broadening of both the Zn 2p and P 2p peaks was observed for Co-Pi/ZnO after PEC measurements, commensurate with the formation of Zn_3_(PO_4_)_2_ species.[42] At the same sample, the signal for Co 2p is barely observed above the background; this indicates desorption of cobalt from the OEC. In contrast, the XP spectra of Ni-B/ZnO after PEC measurements revealed no obvious changes in positions and intensities of Zn, Ni, and B peaks, which is also in agreement with the recently reported enhanced stability of Ni-protected n-Si photoelectrodes.[31] Overall, this study demonstrates the application of Ni-B not only as an efficient surface water-oxidation catalyst, but also as an effective passivation layer for semiconductor electrodes.

## Conclusion

We have reported a mild and efficient approach to significantly improve the photoelectrochemical performance of 1D ZnO nanowire arrays through optimization of their length and diameter followed by surface modification with cheap, earth-abundant cocatalysts. Optimized bare ZnO nanowires (1400 nm length, 70 nm diameter) exhibit a photocurrent density of 0.62 mA cm^−2^ at 1.0 V and 1.2 mA cm^−2^ at 1.6 V (vs. RHE) under 1 sun illumination. Loading of Co-Pi cocatalyst onto the surface of these optimized ZnO nanowires by photoassisted electrodeposition resulted in a higher steady-state photocurrent (0.75 mA cm^−2^ at 1.0 V vs. RHE) and improved IPCE (72 % at 370 nm) for Co-Pi/ZnO compared to bare ZnO. Furthermore, Ni-B/ZnO exhibited twofold higher steady-state photocurrent density (1.22 mA cm^−2^ at 1.0 V vs. RHE) compared to unmodified ZnO, which resulted in an IPCE of about 90 % at 370 nm. More importantly, a significant cathodic shift in onset potential (by 0.2 V) and potential for saturated photocurrent (by nearly 0.7 V) were observed after modification with Ni-B. The stability of ZnO was improved significantly on introduction of these surface oxidation catalysts, and Ni-B/ZnO exhibited an unprecedented zero loss of photocurrent over a 1 h test period, which demonstrates the dual functionality of Ni-B as a benign water-oxidation catalyst and robust surface-protection layer that can inhibit photocorrosion. The overall enhancement in current is due to efficient hole trapping by the surface cocatalyst and its catalytic effect. Besides, fast electron transfer along highly charge mobile, well aligned ZnO wires to the counter electrode also plays an important role. This simple strategy that dramatically improved the efficiency of ZnO may be applicable to other photoanodes, for example, BiVO_4_, α-Fe_2_O_3_, as well as to those that exhibit poor stability that limits their practical use in water-splitting devices. A potential heterojunction architecture would likely involve the coupling of a visible-light-driven photocatalyst to a ZnO nanowire charge acceptor to achieve efficient photocatalytic water cleavage.

## Experimental Section

### Preparation of ZnO electrodes

ZnO nanowire arrays were fabricated on FTO glass substrates from ZnO seeds by a hydrothermal (hydrolysis/condensation) method described by Greene et al.[22] Firstly, ZnO seed crystals were deposited onto FTO glass (TEC 15, Pilkington NSG) by spray pyrolysis (nozzle size 2 cm^2^, at a distance of 15 cm) from a solution containing 0.005 m zinc acetate dihydrate (98 %, Aldrich) in ethanol followed by an annealing process in air at 350 °C for 20 min. This procedure was repeated twice to obtain a uniform coverage of ZnO seed crystals with density and size similar to those already reported.[22a] Secondly, a precursor solution with 0.025 m Zn(NO)_3_**⋅**6 H_2_O (98 %, Sigma) and hexamethylenetetramine (HMT, ≥99.5 %, Sigma) was heated in an open water bath at 90 °C. In our optimized procedure, ZnO films were synthesized by varying the growth time from 0.5 to 5 h, and then by varying the concentration of the precursor solution from 0.025 to 0.01 m in intervals of 0.025 m. The concentration ratio of Zn(NO)_3_**⋅**6 H_2_O and HMT was kept constant at 1:1 while varying the concentration of precursor.

Both Co-Pi and Ni-B catalysts were deposited onto optimized ZnO films by photoassisted electrodeposition.[27–28] A three-electrode system with as-prepared ZnO films as working electrode, Ag/AgCl as reference electrode, and a Pt mesh as counter electrode were used for the photoassisted electrodeposition process. Co-Pi/ZnO and Ni-B/ZnO junctions were synthesized by applying a constant potential of 0.4 V (vs. Ag/AgCl) in a solution of 0.5 mm cobalt nitrate containing 0.1 m potassium phosphate buffer at pH 7 and a solution of 0.1 m potassium borate at pH 9.2 containing 1 mm Ni(NO_3_)_2_, respectively, for 600 s under AM 1.5G light (100 mW cm^−2^) illumination.

### Characterization

X-ray diffraction (XRD) was carried out on a Bruker D8 Advance X-ray diffractometer (40 kV, 30 mA) with Cu_Kα_ radiation (*λ*=1.54 Å) equipped with a PSD LynxEye silicon-strip detector. UV/Vis spectra were obtained on a Shimadzu UV-2550 UV/Vis spectrometer. The bandgap energy was estimated by using Equation (1)


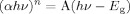
1

where *α* is the absorption coefficient, *h*ν the photon energy (*h* is Planck’s constant, *ν* is the frequency), *n* a constant with a value of 2 for direct-bandgap semiconductors, *A* a proportionality constant related to the material, and *E*_g_ the bandgap energy. The morphologies of the samples were studied on a Jeol JSM-7401F scanning electron microscope. High-resolution XPS was performed by using a Thermo Scientific K-alpha photoelectron spectrometer with monochromatic Al_Kα_ radiation; peak positions were calibrated to carbon (284.5 eV) and plotted with the CasaXPS software.

### PEC measurements

The PEC measurements were conducted in a three-electrode cell equipped with a quartz window and potentiostat (Ivium technology). As-prepared films were used as the working electrode. A Pt mesh and Ag/AgCl were used as a counter electrode and reference electrode, respectively. The scan speed was 20 mV s^−1^ between −0.4 and 1.0 V (vs. Ag/AgCl) All measurements were carried out with a Ag/AgCl (3 m KCl) reference electrode, but results reported in this study are presented against the reversible hydrogen electrode (RHE) for ease of comparison with the H_2_ and O_2_ redox levels and with other literature reports that used electrolytes with different pH. Thus electrode potentials were converted to the RHE scale by using Equation (2).


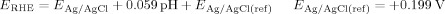
2

The electrolyte was a 0.2 m aqueous solution of Na_2_SO_4_ with 0.1 m phosphate buffer (pH 7) or 0.1 m potassium borate solution (pH 9.2). All electrolytes were purged with argon for 10 min to remove dissolved O_2_ before PEC measurement. A 150 W xenon lamp (Newport, USA) equipped with an AM 1.5G filter was used to irradiate the ZnO electrodes from the front side and was calibrated to 1 sun illumination (100 mW cm^−2^) by using a photodiode.

For IPCE measurements, monochromatic light was generated by using a monochromator and the resultant photocurrent was recorded for wavelengths between 350 nm and 440 nm. The light intensity was measured with a silicon photodiode and a Newport Optical Meter (Model 1918-R). IPCE was calculated by using Equation (3)


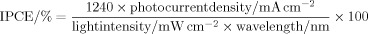
3

The ABPE *η* was estimated by using Equation (4)[43, 44] [Disp-formula m4]


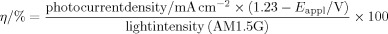
4

## Supporting Information

As a service to our authors and readers, this journal provides supporting information supplied by the authors. Such materials are peer reviewed and may be re-organized for online delivery, but are not copy-edited or typeset. Technical support issues arising from supporting information (other than missing files) should be addressed to the authors.

miscellaneous_information
