# Supplementary material for: Earth-Abundant Oxygen Evolution Catalysts Coupled onto ZnO Nanowire Arrays for Efficient Photoelectrochemical Water Cleavage
Source: Chemistry. 2014 Aug 22;20(40):12954–61. doi: 10.1002/chem.201403067 (PMC4517101; doi:10.1002/chem.201403067)
Supplement: Supplementary file 1 — miscellaneous_information [file chem0020-12954-sd1.pdf]

CHEMISTRY
A European Journal

Supporting Information

© Copyright Wiley-VCH Verlag GmbH & Co. KGaA, 69451 Weinheim, 2014

Earth-Abundant Oxygen Evolution Catalysts Coupled onto ZnO Nanowire Arrays for Efficient Photoelectrochemical Water Cleavage

Chaoran Jiang,[a] Savio J. A. Moniz,[a] Majeda Khraisheh,[b] and Junwang Tang*[a]

chem_201403067_sm_miscellaneous_information.pdf

Supporting Information

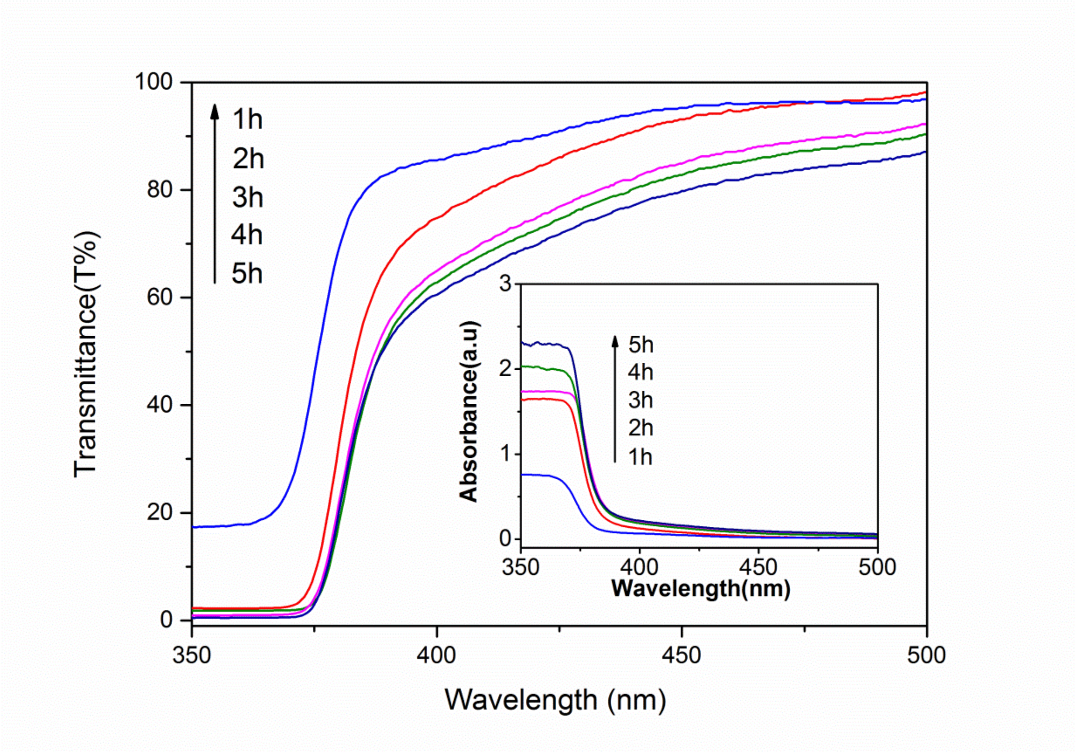

Figure S1: UV-Vis transmittance spectra of ZnO nanowires grown at 90°C (0.025M precursor concentration) as a function of reaction time. (Insert shows the corresponding UV-Vis absorption spectra)

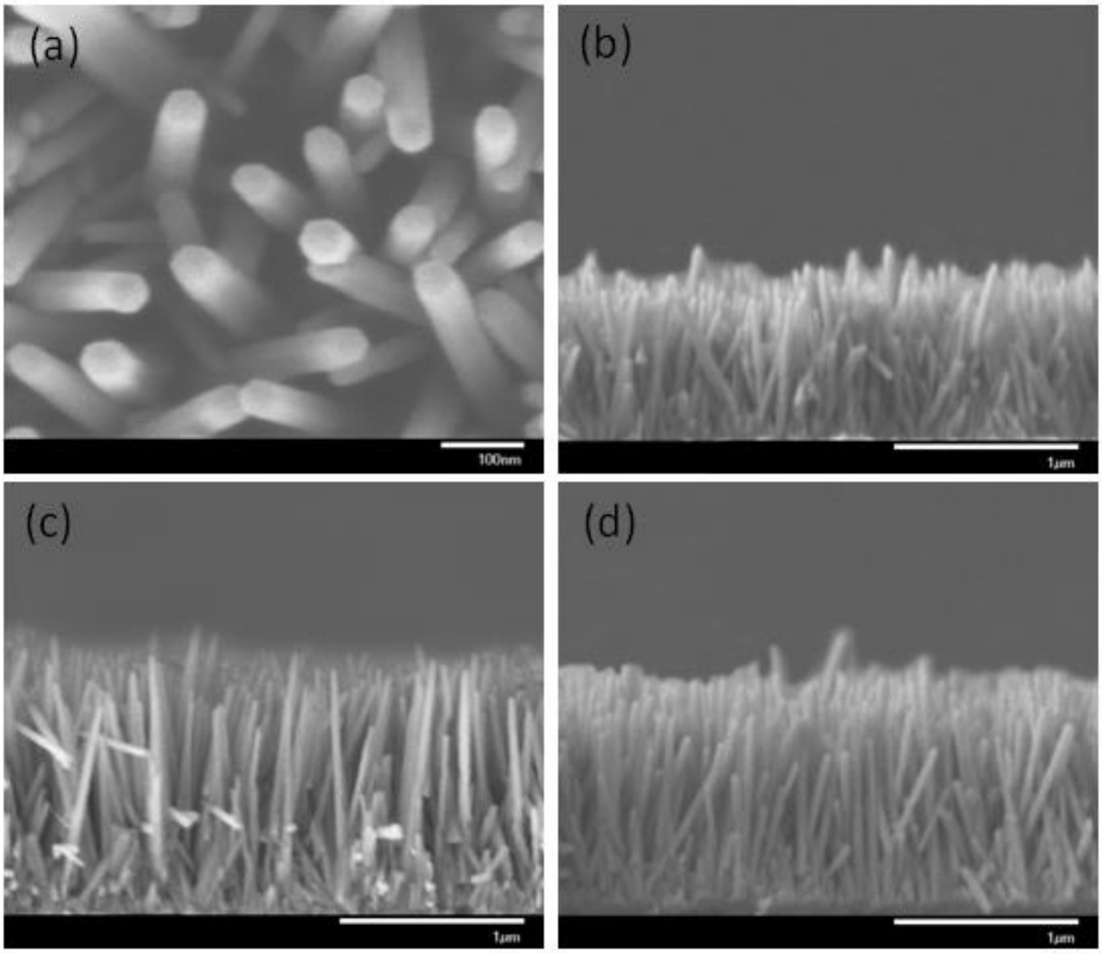

Figure S2: SEM images of ZnO nanowire arrays grown by hydrolysis-condensation reaction with 0.025M precursor concentration at 90°C for different growth times (a) Top view of ZnO wires with reaction time of 4h; (b-d): Side-on view of 3h, 4h, 5h grown ZnO nanowires, respectively.

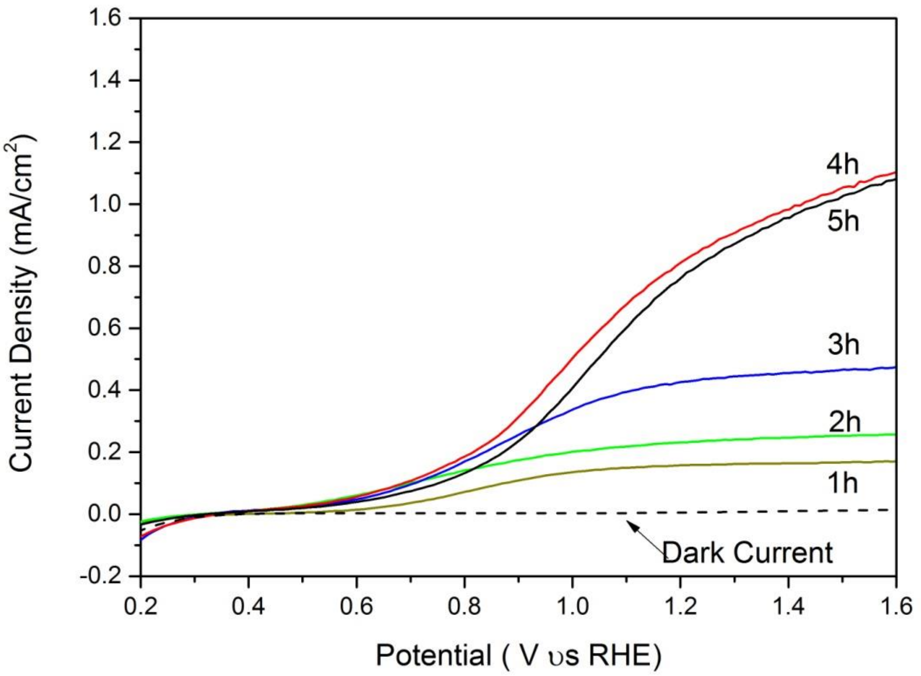

Figure S3: I-V curves measured in a 0.2M Na_2SO_4 solution with phosphate buffer (pH=7) for ZnO films prepared at 90°C with 0.025M precursor concentration for varying reaction time; Dark Scan was indicted by dashed line.

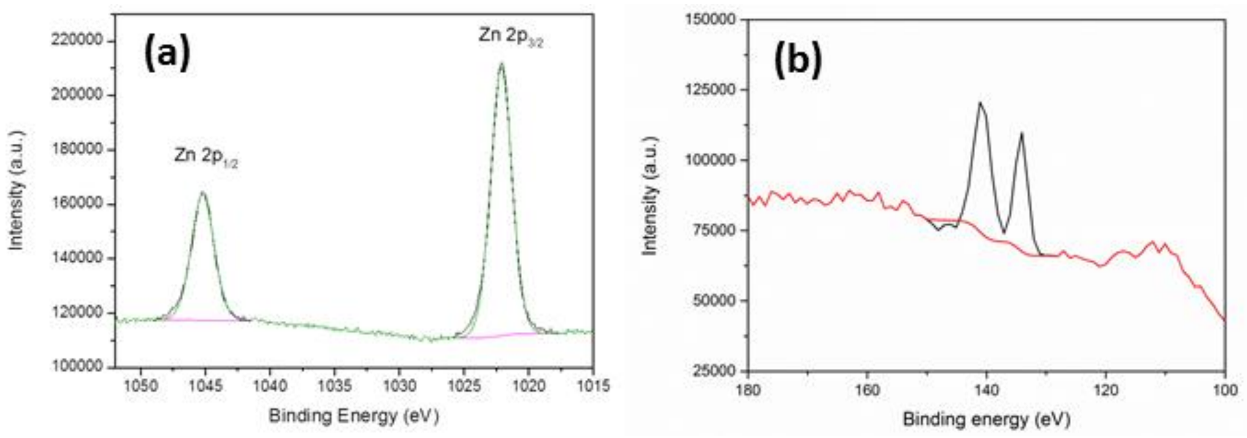

Figure S4: (a) XPS spectrum of Zn 2p; (b) XPS spectrum of bare ZnO nanowires showing contribution from either Zn 3s and/or Sn 4s signals.

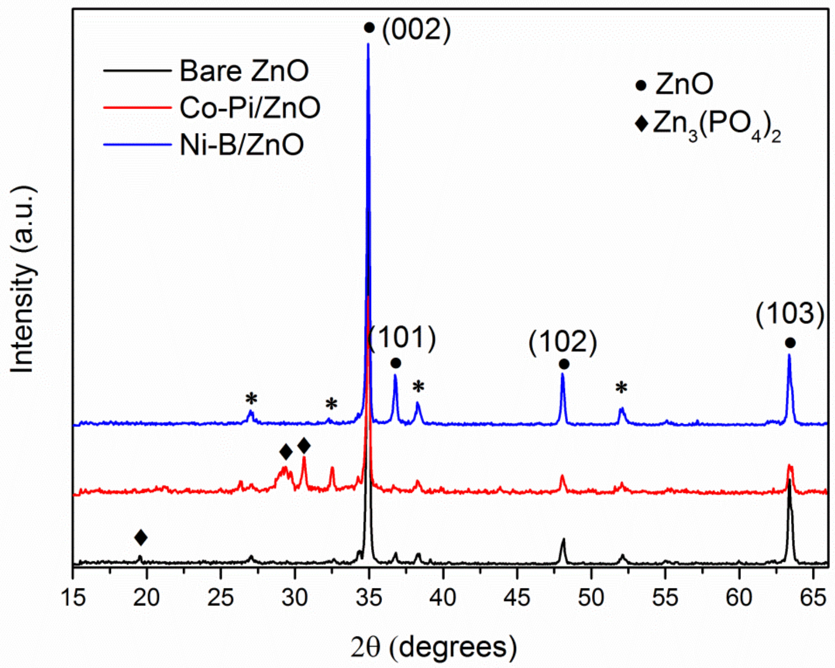

Figure S5: XRD patterns of ZnO nanowire arrays after 1h PEC measurement, (∗) corresponds to the peak of SnO_2 (FTO substrate).

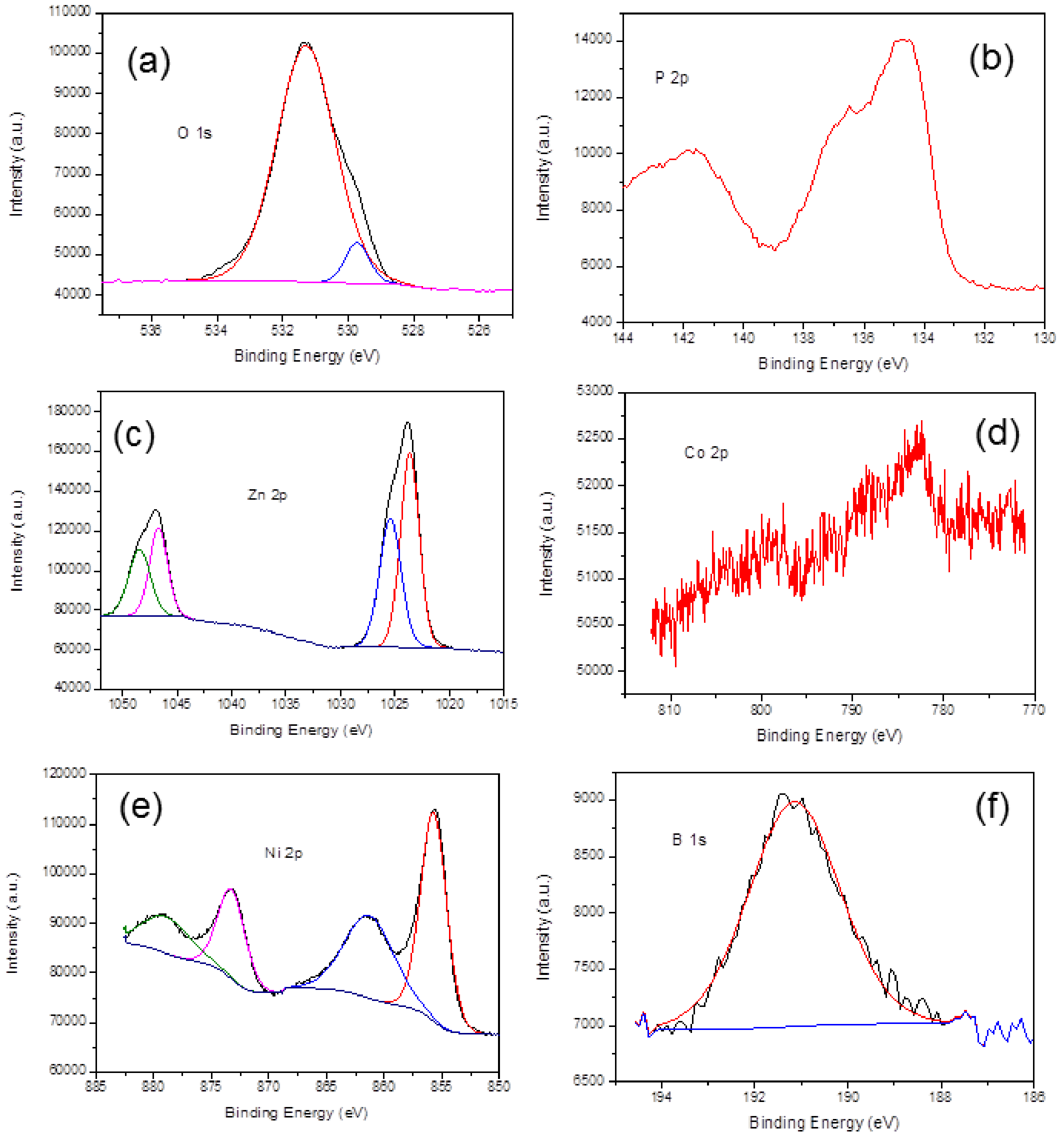

Figure S6: XPS spectra of ZnO samples after PEC measurements: (a) O 1s and (b) P 2p regions of bare ZnO nanowires indicating additional $Zn_3(PO)_4$ formation, (c) Zn 2p and (d) Co 2p regions of Co-Pi/ZnO revealing distortion of Zn peaks and removal of cobalt species, (e) Ni 2p and (f) B 1s regions revealing no change in sample and excellent stability.
